# Supplementary material for: Data-driven drug-induced QT prolongation surveillance using adverse reaction signals derived from 12-lead and continuous electrocardiogram data
Source: PLoS One. 2022 Jan 31;17(1):e0263117. doi: 10.1371/journal.pone.0263117 (PMC8803188; doi:10.1371/journal.pone.0263117)
Supplement: S1 Table — Rank 1 drugs are candidate drugs with unknown QT prolongation risk in the case-control study. The drugs in other ranks were analyzed to validate the study. (DOCX) [file pone.0263117.s001.docx]

**S1 Table. Complete drug list for each drug rank used in counting the frequency of drug use for each drug ranking group within seven days before the ECG measurement date. Rank 1 drugs are candidate drugs with unknown QT prolongation risk in the case-control study. The drugs in other ranks were analyzed to validate the study.**

|  | Drug names | | |
| --- | --- | --- | --- |
| Drugs in rank 1 (unknown risk of QT prolongation) | | | |
|  | Vasopressin | Cefotetan | Thiopental |
|  | somatostatin | Methylolcephalexin | Flumanezil |
|  | Etomidate | Gabexate | Afloqualone |
|  | Methylergometrine | Biphenyldimethyldicarboxylate | Dexamethasone |
|  | Lorazepam | Atropine | Polaprezinc |
|  | Vecuronium Bromide | Nicorandil | Ketamine |
|  | Hydrocortisone | Bisoprolol | Cefoxitin |
|  | Ceftriaxone | Phloroglucinol | Fentanyl |
|  | Ipratropium Bromide | Ulinastatin | Bisacodyl |
|  | Levetiracetam | Scopolamine | Ketorolac |
|  | Perindopril | Cefazolin | Protamine |
|  | Labetalol | Amikacin | Mg dimecrotate |
|  | Ceftazidime | Dioctahedral smectite | Desflurane |
|  | Hydralazine | Levothyroxine | Diltiazem |
|  | Insulin(aspart) | Cefotaxime | Clonazepam |
|  | Rosuvastatin | Aminophylline | Benzydamine |
|  | Carvedilol | Sugammadex | Zolpidem |
|  | Ticagrelor | Sucralfate | Digoxin |
|  | Morphine | Dimenhydrinate | Candesartan |
|  | Captopril | Trimetazidine | Cefmetazole |
|  | Tiotropium | Cimetropium | Rebamipide |
|  | Spironolactone | Verapamil | Olmesartan |
|  | Chlorpheniramine | Metoprolol | Mosapride |
|  | Isosorbide Dinitrate | Cefpodoxime | Tamsulosin |
|  | Cefepime | Glimepiride | Fexofenadine |
|  | Insulin(glulisine) | Telmisartan | Celecoxib |
|  | Levocloperastine | Sufentanil | Choline alfoscerate |
|  | Erdosteine | ranitidine | Oxycodone |
|  | Budesonide | Netilmicin | Tiropramide |
|  | Clopidogrel | Insulin(Glargine) | Pregabalin |
|  | Ibuprofen | Cefotiam | Diomagnate |
|  | Theobromine | Diclofenac | Amlodipine besylate |
|  | Enoxaparin | Acetaminophen | Hyoscin |
|  | Teicoplanin | Flomoxef | Prednisolone |
|  | Pyridoxine | Guaiazulene | Trimebutin maleate |
|  | Remifentanil | Codeine Phosphate | Ramosetron |
|  | Midazolam | Aspirin | Megestrol |
|  | Propranolol | Alprazolam | Ginkgo biloba |
|  | Phenytoin | Metformin | Naproxen |
|  | Milrinone | Acebrophylline | Zaltoprofen |
|  | Ramipril | Levocetirizine | Teprenone |
|  | Nifedipine | Theophylline | Irbesartan |
|  | Warfarin | Benproperine | Doxazosin |
|  | Propacetamol | Nefopam | Pethidine |
|  | Clindamycin | Ceftizoxime | Almagate |
|  | Meropenem | Diazepam | Nimesulide |
|  | Zipepro | Gallamine triethiodide | Pyridostigmine |
|  | Nebivolol | Insulin(Human) | Acyclovir |
|  | Levodropropizine | Atorvastatin | Alfentanil |
|  | Lactitol | Lafutidine | Glycopyrrolate |
|  | Valproate | Rocuronium Bromide | megestrol acetate |
|  | Isosorbide Mononitrate | Losartan | Itopride |
|  | Vancomycin | Entecavir | Gabapentin |
|  | Dried Ivy leaf | Cefazedone | Simethicone |
|  | Cefpiramide | Methylprednisolone | Mepivacaine |
|  | Nimodipine | Ticlodipine |  |
| Drugs in rank 2 (conditional risk of QT prolongation) | | |  |
|  | Esomeprazole | Lansoprazole | Cimetidine |
|  | Famotidine | Metronidazole | Hydrochlorothiazide |
|  | Torasemide | Furosemide | Omeprazole |
|  | Loperamide | Pantoprazole | HydroxyChloroquine |
|  | Quetiapine | Metoclopramide | Trazodonel |
| Drugs in rank 3 (possible risk of QT prolongation) | | |  |
|  | Dexmedetomidine | Ofloxacin | Oxytocin |
|  | Nicardipine | Tramadol | Nortriptyline |
|  | Granisetron | Palonosetron |  |
| Drugs in rank 4 (known risk of QT prolongation) | | |  |
|  | Amiodarone | Cilostazol | Propofol |
|  | Terlipressin | Clarithromycin | Domperidone |
|  | Haloperidol | Moxifloxacin | Roxithromycin |
|  | Azithromycin | Sevoflurane | Levosulpiride |
|  | Ondansetron | Ciprofloxacin |  |
