# Supplementary material for: Data-driven drug-induced QT prolongation surveillance using adverse reaction signals derived from 12-lead and continuous electrocardiogram data
Source: PLoS One. 2022 Jan 31;17(1):e0263117. doi: 10.1371/journal.pone.0263117 (PMC8803188; doi:10.1371/journal.pone.0263117)
Supplement: S2 Table — The QT drug list is based on the QT risk drug list provided in CredibleMeds.org. (DOCX) [file pone.0263117.s002.docx]

**S2 Table. Complete list of the comorbidities and drugs used in the propensity score matching process in survival analysis. The QT drug list is based on the QT risk drug list provided in CredibleMeds.org.**

|  | details |
| --- | --- |
| Comorbidities | Bradycardia​ |
|  | Acute Myocardial Ischemia​ |
|  | Stroke |
|  | Congenital Heart Failure |
|  | Hemodialysis ​(CRF) |
|  | Hypothyroidism​ |
|  | Hypoglycemia​ |
|  | Liver Failure (cirrhosis)​ |
|  | Pulmonary Embolism​ |
|  | Systemic Lupus Erythematosus​ |
|  | Sepsis​ |
|  | Rheumatoid Arthritis​ |
| QT risk drugs | ciprofloxacin |
|  | dopamine |
|  | ephedrine |
|  | epinephrine |
|  | famotidine |
|  | metoclopramide |
|  | metronidazole |
|  | nicardipine |
|  | norepinephrine |
|  | ofloxacin |
|  | pantoprazole |
|  | phenylephrine |
|  | piperacillin |
|  | propofol |
|  | quetiapine |
|  | salbutamol |
|  | tramadol |
|  | furosemide |
