# Supplementary material for: Data-driven drug-induced QT prolongation surveillance using adverse reaction signals derived from 12-lead and continuous electrocardiogram data
Source: PLoS One. 2022 Jan 31;17(1):e0263117. doi: 10.1371/journal.pone.0263117 (PMC8803188; doi:10.1371/journal.pone.0263117)
Supplement: S3 Table — The candidate drug list is the list of drugs in rank 1; the drugs in other ranks were analyzed to validate the study. The drugs in each rank were used to count the drug use frequency of each rank. (DOCX) [file pone.0263117.s003.docx]

**S3 Table. Complete results for all drugs in each of the four drug ranks. The candidate drug list is the list of drugs in rank 1; the drugs in other ranks were analyzed to validate the study. The drugs in each rank were used to count the drug use frequency of each rank.**

|  |  | OR | CI (95%) | *p*-value |
| --- | --- | --- | --- | --- |
| Drugs in rank 1 | |  |  |  |
|  | Vasopressin | 2.05 | 1.97-2.13 | <0.001 |
|  | somatostatin | 1.94 | 1.77-2.11 | <0.001 |
|  | Etomidate | 1.81 | 1.77-1.85 | <0.001 |
|  | Methylergometrine | 1.80 | 1.68-1.92 | <0.001 |
|  | Lorazepam | 1.79 | 1.74-1.84 | <0.001 |
|  | Vecuronium Bromide | 1.68 | 1.63-1.73 | <0.001 |
|  | Hydrocortisone | 1.67 | 1.56-1.78 | <0.001 |
|  | Ceftriaxone | 1.65 | 1.61-1.69 | <0.001 |
|  | Ipratropium Bromide | 1.64 | 1.6-1.68 | <0.001 |
|  | Levetiracetam | 1.64 | 1.58-1.7 | <0.001 |
|  | Perindopril | 1.55 | 1.48-1.62 | <0.001 |
|  | Labetalol | 1.55 | 1.49-1.61 | <0.001 |
|  | Ceftazidime | 1.52 | 1.42-1.62 | <0.001 |
|  | Hydralazine | 1.49 | 1.36-1.62 | <0.001 |
|  | Insulin(aspart) | 1.35 | 1.17-1.53 | 0.036 |
|  | Rosuvastatin | 1.35 | 1.31-1.39 | <0.001 |
|  | Carvedilol | 1.34 | 1.29-1.39 | <0.001 |
|  | Ticagrelor | 1.32 | 1.22-1.42 | 0.003 |
|  | Morphine | 1.32 | 1.26-1.38 | <0.001 |
|  | Captopril | 1.31 | 1.21-1.41 | 0.002 |
|  | Tiotropium | 1.31 | 1.17-1.45 | 0.020 |
|  | Spironolactone | 1.31 | 1.25-1.37 | <0.001 |
|  | Chlorpheniramine | 1.31 | 1.29-1.33 | <0.001 |
|  | Isosorbide Dinitrate | 1.30 | 1.27-1.33 | <0.001 |
|  | Cefepime | 1.27 | 1.09-1.45 | 0.072 |
|  | Insulin(glulisine) | 1.27 | 1.06-1.48 | 0.100 |
|  | Levocloperastine | 1.25 | 1.17-1.33 | 0.002 |
|  | Erdosteine | 1.25 | 1.13-1.37 | 0.023 |
|  | Budesonide | 1.25 | 1.12-1.38 | 0.035 |
|  | Clopidogrel | 1.25 | 1.22-1.28 | <0.001 |
|  | Ibuprofen | 1.24 | 1.17-1.31 | 0.001 |
|  | Theobromine | 1.23 | 1.15-1.31 | 0.004 |
|  | Enoxaparin | 1.22 | 1.09-1.35 | 0.044 |
|  | Teicoplanin | 1.21 | 1.06-1.36 | 0.076 |
|  | Pyridoxine | 1.19 | 1.04-1.34 | 0.100 |
|  | Remifentanil | 1.18 | 1.13-1.23 | <0.001 |
|  | Midazolam | 1.18 | 1.09-1.27 | 0.024 |
|  | Propranolol | 1.18 | 1.05-1.31 | 0.021 |
|  | Phenytoin | 1.18 | 0.99-1.37 | 0.080 |
|  | Milrinone | 1.18 | 1.13-1.21 | 0.156 |
|  | Ramipril | 1.17 | 1.11-1.23 | <0.001 |
|  | Nifedipine | 1.17 | 1.03-1.31 | <0.001 |
|  | Warfarin | 1.17 | 1.15-1.19 | 0.100 |
|  | Propacetamol | 1.17 | 1.09-1.23 | <0.001 |
|  | Clindamycin | 1.16 | 1.05-1.27 | 0.003 |
|  | Meropenem | 1.16 | 1.05-1.27 | 0.064 |
|  | Zipepro | 1.16 | 1.03-1.29 | 0.072 |
|  | Nebivolol | 1.16 | 1.05-1.25 | 0.096 |
|  | Levodropropizine | 1.15 | 1.02-1.28 | 0.060 |
|  | Lactitol | 1.15 | 1.07-1.21 | 0.112 |
|  | Valproate | 1.14 | 1.01-1.27 | 0.020 |
|  | Isosorbide Mononitrate | 1.14 | 1.05-1.21 | 0.120 |
|  | Vancomycin | 1.13 | 1.02-1.24 | 0.048 |
|  | Dried Ivy leaf | 1.13 | 1.05-1.19 | 0.108 |
|  | Cefpiramide | 1.12 | 1.05-1.19 | 0.023 |
|  | Nimodipine | 1.12 | 1.06-1.16 | 0.048 |
|  | Cefotetan | 1.11 | 1.03-1.19 | 0.010 |
|  | Methylolcephalexin | 1.11 | 1.01-1.21 | 0.088 |
|  | Gabexate | 1.11 | 1-1.22 | 0.124 |
|  | Biphenyldimethyldicarboxylate | 1.11 | 1.05-1.15 | 0.144 |
|  | Atropine | 1.10 | 1.02-1.18 | 0.027 |
|  | Nicorandil | 1.10 | 1.02-1.18 | 0.096 |
|  | Bisoprolol | 1.10 | 1.04-1.14 | 0.112 |
|  | Phloroglucinol | 1.09 | 1.02-1.16 | 0.055 |
|  | Ulinastatin | 1.09 | 1.01-1.17 | 0.092 |
|  | Scopolamine | 1.09 | 1.01-1.17 | 0.096 |
|  | Cefazolin | 1.09 | 0.99-1.19 | 0.112 |
|  | Amikacin | 1.09 | 1.02-1.14 | 0.164 |
|  | Dioctahedral smectite | 1.08 | 0.95-1.21 | 0.076 |
|  | Levothyroxine | 1.08 | 1.03-1.11 | 0.220 |
|  | Cefotaxime | 1.07 | 0.99-1.15 | 0.050 |
|  | Aminophylline | 1.07 | 0.97-1.17 | 0.156 |
|  | Sugammadex | 1.07 | 1.02-1.1 | 0.204 |
|  | Sucralfate | 1.06 | 1.02-1.1 | 0.052 |
|  | Dimenhydrinate | 1.06 | 0.95-1.17 | 0.068 |
|  | Trimetazidine | 1.06 | 1.02-1.09 | 0.236 |
|  | Cimetropium | 1.05 | 0.97-1.13 | 0.068 |
|  | Verapamil | 1.05 | 0.46-1.6 | 0.220 |
|  | Metoprolol | 1.03 | 0.97-1.09 | 0.228 |
|  | Cefpodoxime | 1.03 | 0.96-1.1 | 0.252 |
|  | Glimepiride | 1.03 | 0.94-1.12 | 0.288 |
|  | Telmisartan | 1.03 | 0.9-1.16 | 0.292 |
|  | Sufentanil | 1.03 | 1-1.04 | 0.320 |
|  | ranitidine | 1.02 | 0.32-1.72 | 0.136 |
|  | Netilmicin | 1.02 | 0.89-1.13 | 0.280 |
|  | Insulin(Glargine) | 1.01 | 0.18-1.84 | 0.360 |
|  | Cefotiam | 1.01 | 0.91-1.09 | 0.380 |
|  | Diclofenac | 1.00 | 0.98-1.02 | 0.400 |
|  | Acetaminophen | 1.00 | 0.94-1.06 | 0.380 |
|  | Flomoxef | 1.00 | 0.88-1.1 | 0.392 |
|  | Guaiazulene | 0.99 | 0.92-1.05 | 0.384 |
|  | Codeine Phosphate | 0.98 | 0.95-1.01 | 0.316 |
|  | Aspirin | 0.98 | 0.94-1.02 | 0.200 |
|  | Alprazolam | 0.98 | 0.89-1.06 | 0.248 |
|  | Metformin | 0.98 | 0.87-1.09 | 0.308 |
|  | Acebrophylline | 0.98 | 0.87-1.07 | 0.328 |
|  | Levocetirizine | 0.97 | 0.88-1.05 | 0.292 |
|  | Theophylline | 0.96 | 0.86-1.07 | 0.264 |
|  | Benproperine | 0.96 | 0.91-1.01 | 0.284 |
|  | Nefopam | 0.96 | 0.87-1.04 | 0.156 |
|  | Ceftizoxime | 0.95 | 0.9-0.99 | 0.232 |
|  | Diazepam | 0.95 | 0.89-1 | 0.092 |
|  | Gallamine triethiodide | 0.95 | 0.89-1 | 0.128 |
|  | Insulin(Human) | 0.95 | 0.89-1 | 0.132 |
|  | Atorvastatin | 0.95 | 0.82-1.06 | 0.132 |
|  | Lafutidine | 0.94 | 0.9-0.98 | 0.248 |
|  | Rocuronium Bromide | 0.94 | 0.85-1.01 | 0.044 |
|  | Losartan | 0.93 | 0.78-1.09 | 0.148 |
|  | Entecavir | 0.93 | 0.17-1.66 | 0.256 |
|  | Cefazedone | 0.92 | 0.85-0.98 | 0.100 |
|  | Methylprednisolone | 0.92 | 0.83-0.99 | 0.068 |
|  | Ticlodipine | 0.91 | 0.87-0.95 | 0.104 |
|  | Thiopental | 0.91 | 0.79-1.03 | 0.006 |
|  | Flumanezil | 0.91 | 0.85-0.96 | 0.172 |
|  | Afloqualone | 0.91 | 0.87-0.95 | 0.031 |
|  | Dexamethasone | 0.91 | 0.82-1 | 0.006 |
|  | Polaprezinc | 0.91 | 0.77-1.03 | 0.112 |
|  | Ketamine | 0.90 | 0.79-1 | 0.164 |
|  | Cefoxitin | 0.90 | 0.87-0.92 | 0.120 |
|  | Fentanyl | 0.89 | 0.81-0.97 | <0.001 |
|  | Bisacodyl | 0.89 | 0.87-0.91 | 0.068 |
|  | Ketorolac | 0.89 | 0.74-1.04 | <0.001 |
|  | Protamine | 0.89 | 0.86-0.92 | 0.168 |
|  | Mg dimecrotate | 0.89 | 0.81-0.97 | 0.012 |
|  | Desflurane | 0.89 | 0.83-0.94 | 0.052 |
|  | Diltiazem | 0.88 | 0.79-0.95 | 0.003 |
|  | Clonazepam | 0.87 | 0.79-0.95 | 0.032 |
|  | Benzydamine | 0.87 | 0.74-0.99 | 0.035 |
|  | Zolpidem | 0.87 | 0.79-0.92 | 0.096 |
|  | Digoxin | 0.86 | 0.76-0.95 | 0.007 |
|  | Candesartan | 0.86 | 0.74-0.96 | 0.040 |
|  | Cefmetazole | 0.85 | 0.8-0.89 | 0.060 |
|  | Rebamipide | 0.84 | 0.7-0.97 | <0.001 |
|  | Olmesartan | 0.83 | 0.77-0.89 | 0.068 |
|  | Mosapride | 0.83 | 0.74-0.91 | 0.001 |
|  | Tamsulosin | 0.82 | 0.74-0.91 | 0.008 |
|  | Fexofenadine | 0.82 | 0.73-0.91 | 0.008 |
|  | Celecoxib | 0.82 | 0.71-0.92 | 0.021 |
|  | Choline alfoscerate | 0.81 | 0.68-0.94 | 0.044 |
|  | Oxycodone | 0.81 | 0.72-0.91 | 0.011 |
|  | Tiropramide | 0.81 | 0.76-0.85 | <0.001 |
|  | Pregabalin | 0.80 | 0.69-0.91 | 0.016 |
|  | Diomagnate | 0.80 | 0.72-0.88 | 0.003 |
|  | Amlodipine besylate | 0.80 | 0.75-0.84 | <0.001 |
|  | Hyoscin | 0.79 | 0.69-0.89 | 0.007 |
|  | Prednisolone | 0.79 | 0.73-0.84 | <0.001 |
|  | Trimebutin maleate | 0.79 | 0.73-0.84 | <0.001 |
|  | Ramosetron | 0.76 | 0.71-0.82 | <0.001 |
|  | Megestrol | 0.76 | 0.66-0.87 | 0.036 |
|  | Ginkgo biloba | 0.76 | 0.68-0.84 | 0.002 |
|  | Naproxen | 0.76 | 0.67-0.86 | 0.002 |
|  | Zaltoprofen | 0.74 | 0.69-0.8 | <0.001 |
|  | Teprenone | 0.74 | 0.66-0.83 | <0.001 |
|  | Irbesartan | 0.72 | 0.61-0.84 | 0.002 |
|  | Doxazosin | 0.70 | 0.57-0.82 | 0.002 |
|  | Pethidine | 0.67 | 0.64-0.71 | <0.001 |
|  | Almagate | 0.66 | 0.56-0.76 | <0.001 |
|  | Nimesulide | 0.65 | 0.54-0.76 | <0.001 |
|  | Pyridostigmine | 0.65 | 0.61-0.69 | <0.001 |
|  | Acyclovir | 0.65 | 0.54-0.75 | <0.001 |
|  | Alfentanil | 0.63 | 0.55-0.7 | <0.001 |
|  | Glycopyrrolate | 0.62 | 0.58-0.66 | <0.001 |
|  | megestrol acetate | 0.59 | 0.46-0.71 | <0.001 |
|  | Itopride | 0.54 | 0.45-0.62 | <0.001 |
|  | Gabapentin | 0.51 | 0.45-0.57 | <0.001 |
|  | Simethicone | 0.51 | 0.43-0.59 | <0.001 |
|  | Mepivacaine | 0.41 | 0.32-0.49 | <0.001 |
| Drugs in rank 2 | |  |  |  |
|  | Esomeprazole | 0.79 | 0.67-0.91 | 0.081 |
|  | Famotidine | 0.94 | 0.9-0.98 | 0.141 |
|  | Torasemide | 0.83 | 0.71-0.95 | 0.149 |
|  | Loperamide | 0.78 | 0.61-0.94 | 0.153 |
|  | Quetiapine | 0.90 | 0.78-1.01 | 0.424 |
|  | Lansoprazole | 0.94 | 0.87-1.02 | 0.504 |
|  | Metronidazole | 1.00 | 0.96-1.03 | 0.940 |
|  | Furosemide | 1.33 | 1.3-1.36 | <0.001 |
|  | Pantoprazole | 1.15 | 1.11-1.19 | <0.001 |
|  | Metoclopramide | 1.10 | 1.08-1.12 | <0.001 |
|  | Cimetidine | 0.69 | 0.66-0.72 | <0.001 |
|  | Hydrochlorothiazide | 0.68 | 0.6-0.76 | <0.001 |
|  | Omeprazole | 0.67 | 0.56-0.78 | <0.001 |
|  | HydroxyChloroquine | 0.63 | 0.49-0.77 | <0.001 |
|  | Trazodonel | 0.50 | 0.37-0.63 | <0.001 |
| Drugs in rank 3 | |  |  |  |
|  | Dexmedetomidine | 1.87 | 1.79-1.95 | <0.001 |
|  | Nicardipine | 1.54 | 1.07-2.01 | <0.001 |
|  | Granisetron | 0.99 | 0.89-1.08 | 0.920 |
|  | Ofloxacin | 0.95 | 0.9-1.01 | 0.426 |
|  | Tramadol | 0.94 | 0.92-0.96 | 0.013 |
|  | Palonosetron | 0.77 | 0.7-0.84 | 0.005 |
|  | Oxytocin | 0.63 | 0.51-0.75 | 0.004 |
|  | Nortriptyline | 0.36 | 0.25-0.46 | <0.001 |
| Drugs in rank 4 | |  |  |  |
|  | Amiodarone | 2.28 | 2.19-2.37 | <0.001 |
|  | Terlipressin | 2.20 | 2.07-2.33 | <0.001 |
|  | Haloperidol | 1.65 | 1.57-1.73 | <0.001 |
|  | Azithromycin | 1.53 | 1.41-1.65 | <0.001 |
|  | Ondansetron | 1.35 | 1.2-1.5 | 0.041 |
|  | Cilostazol | 1.30 | 1.22-1.38 | <0.001 |
|  | Clarithromycin | 1.26 | 1.2-1.32 | <0.001 |
|  | Moxifloxacin | 1.23 | 1.14-1.32 | 0.031 |
|  | Sevoflurane | 1.09 | 1.05-1.13 | 0.041 |
|  | Ciprofloxacin | 0.91 | 0.85-0.96 | 0.076 |
|  | Propofol | 0.90 | 0.87-0.94 | 0.007 |
|  | Domperidone | 0.90 | 0.82-0.97 | 0.172 |
|  | Roxithromycin | 0.90 | 0.81-0.99 | 0.220 |
|  | Levosulpiride | 0.73 | 0.67-0.78 | <0.001 |
