# Supplementary material for: Data-driven drug-induced QT prolongation surveillance using adverse reaction signals derived from 12-lead and continuous electrocardiogram data
Source: PLoS One. 2022 Jan 31;17(1):e0263117. doi: 10.1371/journal.pone.0263117 (PMC8803188; doi:10.1371/journal.pone.0263117)
Supplement: S4 Table — (DOCX) [file pone.0263117.s004.docx]

**S4 Table. Complete analysis results of the 78 drugs considered in the survival analysis based on the continuous ECG database.**

|  |  | HR | CI | *p*-value |
| --- | --- | --- | --- | --- |
| known risk of QT | |  |  |  |
|  | ciprofloxacin | 3.5887 | 1.79-7.19 | <0.001 |
|  | dopamine | 2.9062 | 1.72-4.92 | <0.001 |
|  | ephedrine | 1.6735 | 0.8-3.52 | 0.325 |
|  | epinephrine | 4.2723 | 2.75-6.65 | <0.001 |
|  | famotidine | 0.9281 | 0.74-1.17 | 0.742 |
|  | metoclopramide | 1.5626 | 1.03-2.36 | 0.119 |
|  | metronidazole | 3.4689 | 2.63-4.58 | <0.001 |
|  | nicardipine | 1.8991 | 1.39-2.59 | <0.001 |
|  | norepinephrine | 2.0067 | 1.62-2.48 | <0.001 |
|  | ofloxacin | 2.4631 | 1.32-4.59 | 0.025 |
|  | pantoprazole | 2.568 | 1.91-3.45 | <0.001 |
|  | phenylephrine | 1.6865 | 1.04-2.72 | 0.117 |
|  | piperacillin | 1.6781 | 1.25-2.24 | <0.001 |
|  | propofol | 1.9102 | 1.43-2.55 | <0.001 |
|  | quetiapine | 1.2535 | 0.86-1.84 | 0.412 |
|  | salbutamol | 1.7241 | 1.07-2.78 | 0.099 |
|  | tramadol | 1.6354 | 1.21-2.2 | 0.006 |
|  | furosemide | 2.28 | 1.75-2.81 | <0.001 |
| unknown risk of QT | |  |  |  |
|  | Vasopressin | 1.5 | 1.33-1.65 | 0.024 |
|  | Vecuronium | 1.8 | 1.53-1.99 | 0.021 |
|  | Ranitidine | 1.3 | 1.23-1.46 | 0.080 |
|  | Midazolam | 1.4 | 1.27-1.47 | 0.028 |
|  | Levetiracetam | 1.5 | 1.3-1.72 | <0.001 |
|  | Ipratropium bromide | 1.4 | 1.32-1.48 | <0.001 |
|  | Sucralfate | 1.22 | 1.03 - 1.41 | 0.142 |
|  | nifedipine | 1.33 | 1.16 - 1.5 | 0.008 |
|  | amlodipine | 1.21 | 1.02 - 1.4 | 0.142 |
|  | pyridostigmine | 1.09 | 1.02 - 1.16 | 0.079 |
|  | chlorphenamine | 1.06 | 1.02 - 1.1 | 0.008 |
|  | lorazepam | 1.08 | 1.0 - 1.16 | 0.199 |
|  | tetanus immunoglobulin | 0.97 | 0.92 - 1.02 | 0.279 |
|  | amino acids | 1 | 0.94 - 1.06 | 1.000 |
|  | clopidogrel | 1.11 | 0.98 - 1.24 | 0.254 |
|  | benzydamine | 1.11 | 0.91 - 1.31 | 0.505 |
|  | thiopental | 1.09 | 0.97 - 1.21 | 0.328 |
|  | fentanyl | 1.11 | 0.98 - 1.24 | 0.262 |
|  | antithrombin III | 1 | 0.87 - 1.13 | 1.000 |
|  | metoprolol | 1 | 0.66 - 1.34 | 1.000 |
|  | labetalol | 1 | 0.87 - 1.13 | 1.000 |
|  | gabapentin | 1 | 0.85 - 1.15 | 1.000 |
|  | carvedilol | 1.13 | 0.98 - 1.28 | 0.254 |
|  | glycerol | 1 | 0.76 - 1.24 | 1.000 |
|  | Other cicatrizants | 1.02 | 0.89 - 1.15 | 0.991 |
|  | naproxen | 1 | 0.83 - 1.17 | 1.000 |
|  | dexamethasone | 1.02 | 0.91 - 1.13 | 0.860 |
|  | ceftazidime | 0.87 | 0.76 - 0.98 | 0.079 |
|  | hydrocortisone | 1 | 0.79 - 1.21 | 1.000 |
|  | tranexamic acid | 1.06 | 0.97 - 1.15 | 0.373 |
|  | etomidate | 1.04 | 0.97 - 1.11 | 0.505 |
|  | methylphenidate | 1 | 0.71 - 1.29 | 1.000 |
|  | remifentanil | 1.02 | 0.94 - 1.1 | 0.860 |
|  | vecuronium | 0.98 | 0.9 - 1.06 | 0.793 |
|  | nimodipine | 0.95 | 0.88 - 1.02 | 0.254 |
|  | bisacodyl | 1 | 0.8 - 1.2 | 1.000 |
|  | sodium bicarbonate | 1.06 | 0.99 - 1.13 | 0.193 |
|  | acetylsalicylic acid | 1 | 0.83 - 1.17 | 1.000 |
|  | cisatracurium | 1 | 0.86 - 1.14 | 1.000 |
|  | lactulose | 1 | 0.81 - 1.19 | 1.000 |
|  | heparin | 1.03 | 0.97 - 1.09 | 0.556 |
|  | valproic acid | 1.02 | 0.96 - 1.08 | 0.655 |
|  | mannitol | 1.05 | 1.0 - 1.1 | 0.220 |
|  | enoxaparin | 1 | 0.88 - 1.12 | 1.000 |
|  | magnesium sulfate | 1.06 | 1.01 - 1.11 | 0.135 |
|  | phytomenadione | 0.97 | 0.92 - 1.02 | 0.373 |
|  | potassium chloride | 1.06 | 0.97 - 1.15 | 0.373 |
|  | ketorolac | 0.94 | 0.87 - 1.01 | 0.197 |
|  | insulin (human) | 1.03 | 0.97 - 1.09 | 0.537 |
|  | lidocaine | 1.01 | 0.96 - 1.06 | 0.804 |
|  | fentanyl | 1.04 | 0.99 - 1.09 | 0.254 |
|  | rocuronium bromide | 1.02 | 0.97 - 1.07 | 0.563 |
|  | cefotetan | 1.03 | 0.98 - 1.08 | 0.505 |
|  | ceftriaxone | 1.01 | 0.97 - 1.05 | 0.855 |
|  | paracetamol | 0.96 | 0.91 - 1.01 | 0.279 |
|  | albumin | 1.04 | 1.0 - 1.08 | 0.254 |
|  | vancomycin | 1.1 | 0.97 - 1.23 | 0.279 |
|  | metformin | 1 | 0.36 - 1.64 | 1.000 |
|  | diosmectite | 1 | 0.72 - 1.28 | 1.000 |
|  | acetylcysteine | 0.99 | 0.97 - 1.01 | 0.698 |
